# Trauma context exerts intergenerational effects on child mental health via DNA methylation

**DOI:** 10.1080/15592294.2024.2333654

**Published:** 2024-04-05

**Authors:** Stefanie Pilkay, Andie Riffer, Andrew Carroll

**Affiliations:** a Falk College of Sport and Human Dynamics, School of Social Work, Syracuse University, Syracuse, NY USA; b Jane Addams College of Social Work, University of Illinois at Chicago, Chicago, IL, USA

**Keywords:** DNA methylation, trauma, intergenerational effects, ALSPAC

## Abstract

Many people experience traumatic or negative events, but few develop mental health issues as a result. This study investigated whether newborn DNA methylation (DNAm) previously associated with maternal childhood physical abuse by her father affected the child’s mental health and physical growth, as well as whether it mediated or moderated developmental outcomes. Methods: Study sample (*N* = 903) and data came from Bristol University’s Avon Longitudinal Study of Parents and Children. DNAm was measured in cord blood at birth. DNAm data was preprocessed, normalized, and quality controlled before subsetting to 60 CpG sites of interest from previous research. Linear regression analysis examined newborn DNAm and child development outcome associations. Sobel test examined the mediating relationship between mother’s history of childhood abuse by father, newborn targeted gene DNAm of significant CpG sites, and child’s mental health and physical growth. Moderation analyses examined the interaction effects between the significant CpG sites and mothers’ physical abuse by their fathers on child’s mental health and physical growth. Results: Full cohort analyses showed that newborn DNAm of several different CpG sites associates with separation anxiety, fear, and unhappy/tearful presentations in children aged 6–7 y. Sex-specific associations emerged with boys showing associations with anxiety and fear, and girls showing associations with fear and unhappiness. In boys only, cord blood DNAm mediates the effect of maternal childhood trauma on offspring mental health. No moderation effects emerged. Conclusion: Intergenerational effects of mother’s relationship to her abuser present in newborn DNAm associate with 7-year-old child’s mental health, show sex-specific effects, and newborn DNAm does mediate maternal childhood trauma effects on offspring mental health in early-life.

## Introduction

An appreciable number of people are subjected to traumatic or adverse events during their lifetimes, yet a limited portion of them develop mental health issues as a consequence of these experiences. The disparity between these groups may be discerned via aberrant activity within the hypothalamus-pituitary-adrenal (HPA) axis, along with an amplified production of cortisol and glucocorticoid receptor (GR) alterations that are known to instigate excitotoxicity and neural pruning [1].

Recent scholarly inquiries postulate that the detrimental effects of an excessively active HPA axis could be passed on to the offspring of mothers plagued by chronic stress, thereby inducing susceptibilities to psychopathologies in the newborns, through a process referred to as DNA methylation [1]. DNA methylation, a mechanism that determines gene function and consequently influences mental and physical health outcomes, is implicated in cellular differentiation during foetal development, as well as in modulation of genome function as a response to the environment and early life experiences of an individual [2].

Further, evidence underscores the concept that DNA methylation functions as a lifelong mechanism for genome adaptation [2]. Additionally, studies have illuminated that DNA methylation profiles and psychiatric symptoms in children differ based on socioeconomic conditions, race, and ethnicity [3–5]. A correlation has been observed where increased symptomatology reports are associated with higher salivary DNA methylation profiles indicating greater inflammation and accelerated biological ageing [6].

Moreover, DNA methylation in newborn cord blood has been correlated with modified gene expression, body size, and body composition in childhood [7]. Intriguingly, intergenerational effects have been observed wherein maternal trauma in childhood is associated with DNA methylation in newborns on the day of birth, specifically impacting the BDNF gene, which is responsible for directing the production of brain-derived neurotrophic factors [8].

Recent discoveries propose that the mechanistic role of DNA methylation could potentially function as a mediating and moderating factor between early-life stress and the emergence of psychopathology later in life [4]. Research conducted by Pilkay, Wang, and Nunes identified 33 CpG sites as statistically significant for differential DNA methylation in newborns based on whether their mother had been physically abused by her father in childhood using the same data analysed for this study [9]. The researchers discovered that intergenerational effects on newborn DNA methylation could be observed through an Epigenome-Wide Association Study (EWAS) analysis based on the mother’s relationship to her abuser in childhood. The significant CpG sites were found to play a regulatory role in systems that affect development and mental health. Therefore, the focus of this study was to determine if 1) the newborn DNA methylation linked to mothers who had been physically abused by their fathers was also associated with child developmental outcomes in terms of mental health and physical growth, and 2) if newborn DNA methylation mediated or moderated developmental outcomes.

## Methods

### Variables

#### DNA methylation

DNA methylation was measured in cord blood collected at birth. Using the HumanMethylation450 BeadChip, which assesses >450,000 CpG sites across the genome, DNA methylation from newborn cord blood samples was analysed for each subject. According to the manufacturer’s instructions, 1 g of DNA was processed and hybridized to the HumanMethylation450 BeadChip (Illumina). Using the R package CpGassoc [10], an initial data quality check was undertaken. CpG sites with minimal signal or missing data for more than 10% of samples were removed, as were samples with missing data for more than 5% of CpG sites. Cross-Reactive probes were then eliminated [11]. For each CpG site, beta values (β) were calculated as the ratio of methylated (M) to methylated and unmethylated (M+U) signal: β = M/M+U. Quantile normalization was carried out as described previously [12]. After quality control, the 60 targeted probes identified in previous research were incorporated into subsequent analyses. Covariate information on the newborn’s sex was obtained from hospital birth records. The covariates mPC2 and mPC3 were previously identified as genetic controls for ancestry [13], and the authors elected to follow this covariate protocol in any likelihood that they provide a greater robust control than parent reported ‘race’ according to predefined categories. Cellular heterogeneity (i.e., the proportion of CD8+T, CD4+T, natural killer (NK), B cells, monocytes, and neutrophils) was predicted using the Robust Partial Correlation (RPC) method implemented in Epidish [14] using the reference data reported by Salas, Wiencke, Koestler, Zhang, Christensen, Kelsey [15]. ComBat was used to adjust for batch effects of chip and position [16]. Consent for biological samples has been collected in accordance with the Human Tissue Act (2004).

#### Maternal childhood physical abuser

At the start of participation, mothers’ childhood abuse and demographic information were assessed using a self-reported ‘mother questionnaire.’ Mothers were asked to indicate if they had been physically abused in their childhood, as well as whether the abuser was their ‘mother,’ ‘father,’ or ‘other person.’ The focus of this variable is not the type of abuse itself, but rather the relationship to the abuser, and to maintain model integrity with the previous study from which the targeted CpG sites were identified for this investigation [9] in order to test intergenerational effects on child development. Informed consent for the use of data collected via questionnaires and clinics was obtained from participants following the recommendations of the ALSPAC Ethics and Law Committee at the time.

#### Child mental health and physical growth

A total of seven child outcome variables were tested, with one additional dichotomous variable included for sensitivity analysis only. Child mental health, at age 6–7 y, was measured by indicators of anxiety and depression. Child anxiety was represented by parent and school reports of anxiety symptoms. Parents reported on separation anxiety (any separation anxiety symptoms [0 = no, 1 = yes], number of different symptoms [continuous], separation anxiety symptom score [continuous]), and child’s general anxiety symptom score [continuous]. The dichotomous variable ‘any separation anxiety symptoms’ was used for a sensitivity analysis only to determine how different values of the child’s separation anxiety could affect associations with DNA methylation. Number of different separation anxiety symptoms was included to be an indicator of a greater complexity of separation anxiety experienced by the child as previous research has indicated health associations with the number of different anxiety symptoms [17]. We aimed to determine if differences in DNA methylation associations are present among separation anxiety symptom score and number of different separation anxiety symptoms (previously indicated as a complexity measure of separation anxiety). Child’s school reported on ‘degree to which child had many worries in the past 6 months’ (0 = not true, 1 = somewhat true, 2 = certainly true), and ‘degree to which child had many fears in the past 6 months’ (0 = not true, 1 = somewhat true, 2 = certainly true). Child depression was represented by school reports on ‘degree to which child was often unhappy or tearful in the past 6 months’ (0 = not true, 1 = somewhat true, 2 = certainly true). The school reports of child worries, fears, and unhappy or tearful presentations were selected to target the younger aged child where standardized scale scores were not available but indicated symptoms consistent with the standardized Revised Child Anxiety and Depression Scale normed on children beginning at 8 y of age [18]. The authors elected to choose measures that indicated the variables of interest while maintaining a close timing proximity to the newborn DNA methylation. Child physical growth was measured with adiposity rebound (continuous) calculated by determining the second rise in BMI between ages two to seven y. Adiposity rebound, specifically, was chosen due to the previous study gene ontology findings indicating metabolism as one system associated with the targeted genes in this study that associated with mothers who were abused by their fathers in childhood [9]. Adiposity rebound has been linked to obesity, diabetes, and cardiovascular disease [19], and is predictive of metabolic risk in seven year old children [20]. Adiposity rebound was calculated by visual inspection of an upward trend in body mass index (BMI) after its nadir as previously described [21,22]. To reduce possible measurement error from subjective interpretation, researchers confirmed all consecutive BMI measurements increased after nadir and all increases were equal to or greater than .1 kg/m^2^. Adiposity rebound (AR) was categorically coded (0, 1, 2) to represent very early (before 43 months), early (49 months to before 61 months), and later AR (after 61 months).

### Sample

This study sample and data were obtained from the University of Bristol’s Avon Longitudinal Study of Parents of Children (ALSPAC) [23–25]. The ALSPAC study invited pregnant women residing in Avon, UK, with anticipated delivery dates ranging from 1 April 1991, to 31 December 1992, to participate. A total of 20,248 pregnancies were found to meet the eligibility criteria, and out of those 14,541 pregnancies were initially enrolled. Out of the pregnancies that occurred initially, there were a total of 14,676 developing embryos. This led to 14,062 newborns who were alive at birth and 13,988 children who survived until they reached 1 year of age. The overall sample size for analyses involving data collected after the age of seven is 15,447 pregnancies, which corresponds to 15,658 foetuses. Out of this total, a sum of 14,901 children were still living when they reached the age of one year. Out of the total of 14,541 initial pregnancies, 338 were accounted for by a woman who had already participated in the study with a previous pregnancy. Therefore, the study initially included 14,203 distinct mothers. Due to the subsequent recruitment phases, an additional 630 women who were not initially enrolled have contributed data since their child reached the age of 7. As of September 2021, there are a total of 14,833 distinct women (referred to as G0 mothers) who are registered in ALSPAC. The G0 partners were requested to fill out questionnaires initiated by the mothers at the beginning of the study, but they were not officially registered at that point. A total of 12,113 G0 partners have engaged with the study by either providing data or formally enrolling since its inception in 2010. There are currently 3,807 partners enrolled in the G0 program.

This longitudinal study began in the 1990s and has now accumulated a cohort of three generations. Large cohorts are required to achieve sufficient statistical power and reduce type I error for epigenome-wide analysis (EWAS), but they are expensive and difficult to recruit such a large number of participants [26]. One of the largest DNA methylation cohorts (ALSPAC) is made up of 903 mother/newborn pairs. Furthermore, the large ALSPAC cohort provides an exceptional opportunity to investigate intergenerational trauma effects on a biological level with greater generalizability in order to inform potential novel intervention possibilities for observed generational patterns of trauma and mental health symptoms. Our study sample included 903 mother/newborn couples who provided cord blood samples for DNA methylation profiling. Mothers’ average age at the time of birth was 29.2 y (*SD* = 5.5 y). The newborn cohort is roughly equally divided by sex (Male = 51%), the majority were identified as Caucasians (96%), and the majority were reported to be in the middle socioeconomic class (low income = 6%, middle income = 82%, and high income = 12%). Please note that the study website contains details of all the data that is available through a fully searchable data dictionary and variable search tool available on their website (http://www.bristol.ac.uk/alspac/researchers/our-data/).

### Statistical analysis

The DNA methylation data was preprocessed, normalized, and quality controlled before being subset to include the top 60 CpG sites of interest according to p-values. The analysis initially focused on the complete cohort of children using the standard linear regression models as advised by Mansell, Gorrie-Stone, Bao, Kumari, Schalkwyk, Mill, Hannon [27]. Specifically, an EWAS (epigenome-wide analysis) approach was applied to the targeted 60 CpG sites and to generate the correct multiple comparison control through the False Discovery Rate [28]. This approach treats all phenotype variables of interest as ‘predictors’ and the DNA methylation of the targeted CpG sites as ‘outcomes’ as has previously been conducted [27]. Significant associations are then plotted to accurately interpret the beta coefficients and are subsequently described in the results. The models were adjusted for cell type, ancestry (race) as reported by parents for the child, sex, socioeconomic status, and gestational age, while also accounting for batch effects of chip and position during the quality control phase. Socioeconomic status [29] and gestational age [30] have previously been linked to newborn DNAm and were added as control variables. The significance level was adjusted using the False Discovery Rate [28] modification, while implementing multiple comparison control. The p-values presented for the EWAS studies are adjusted using the False Discovery Rate (FDR) method, with a significance threshold set at *p* < .05. This threshold corresponds to the original p-values being less than .007. The researchers plotted noteworthy relationships. The experiments were repeated with stratification by sex in order to investigate the presence of relationships particular to each sex that will allow for meaningful interpretation of the results. All previously identified variables in the linear regression models were retained, except for the inclusion of sex as a covariate. The technique of pairwise deletion was employed to handle missing data in the phenotypic variables, whereas the DNAm data was found to be full without any missing values.

Mediation and moderation models were chosen for examination because DNAm has been shown to play mediating and moderating roles as both in relation to early-life stress and trauma [4]. The mechanism of effect is not the focus for this investigation, but to discover what, if any, intergenerational mechanism is at play. Continuous child outcome variables were analysed for DNA methylation mediation with the Sobel test. Approaches have been identified to address concerns around categorical outcome variables in mediation models [31]. However, the authors elected to not analyse the categorical child outcome variables (Y in the mediation model) for mediation due to a lack of strong scholarly consensus on how to accurately test these models to achieve trustworthy results [31]. The study employed moderation analyses to examine the relationship between the found CpG site and mothers’ physical abuse by their fathers. Computed interaction terms were utilized for this purpose. The multiple regression models incorporated the interaction term alongside each predictor variable as covariates. Multinomial logistic regression was applied for categorical child outcome variables. In the event that multicollinearity was shown to be inflated, as evidenced by a Variance Inflation Factor (VIF) ≥10, the predictor variables underwent mean centring and were then re-evaluated. Ethical approval for the study was obtained from the ALSPAC Ethics and Law Committee and the Local Research Ethics Committees.

## Results

**Figure 1.**
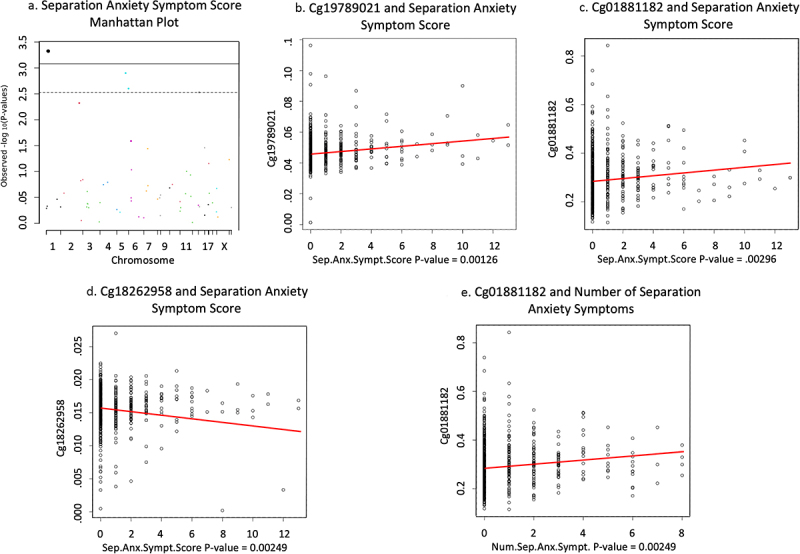
Full cohort anxiety associations.

Increased DNAm of cg01881182 located on the transcription start site 1500 of the ABAT gene is associated with increased number of different separation anxiety symptoms shown in Figure 1 (*n* = 815, B = .041, *t* = 3.697, FDR adjusted *p* = .01, non-adjusted p-value = .0002). DNAm of cg19789021 on the body of GLRX (*n* = 791, B = .017, *t* = 3.239, FDR adjusted *p* = .037, non-adjusted p-value = .001) and cg01881182 on ABAT (*n* = 791, B = .026, *t* = 2.982, FDR adjusted *p* = .044, non-adjusted p-value = .002) positively associated with separation anxiety symptom score (see Figure 1). However, DNAm of cg18262958 on the transcription start site 200 of LMNB1 was negatively associated with separation anxiety symptom score (*n* = 791, B = −.019, *t* = −3.035, FDR adjusted *p* = .044, non-adjusted p-value = .002) shown in Figure 1. Sensitivity analysis of separation anxiety revealed that there is a difference among associations according to how separation anxiety is measured. Specifically, reduced methylation of cg01881182 (ABAT) in newborn cord blood is associated with the presence of any separation anxiety symptoms in seven-year-old children (*n* = 815, B = −.141, *t* = −4.144, FDR adjusted *p* = .002, non-adjusted p-value = 3.81 × 10^−5^) which is the opposite methylation pattern found associated with number of different separation anxiety symptoms.

(a) Manhattan plot of separation anxiety symptom score associations in full cohort. Dots above dotted line are FDR adjusted *p* < .05. (b) Scatterplot of GLRX DNAm association with separation anxiety symptom score in full cohort. (c) Scatterplot of ABAT DNAm association with separation anxiety symptom score in full cohort. (d) Scatterplot of XX DNAm association with separation anxiety symptom score in full cohort. (e) Scatterplot of ABAT DNAm association with number of separation anxiety symptoms in full cohort.

**Figure 2.**
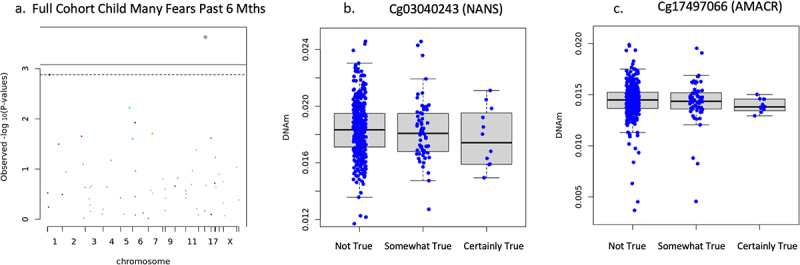
Full cohort child had many fears in past 6 months associations.

**Figure 3.**
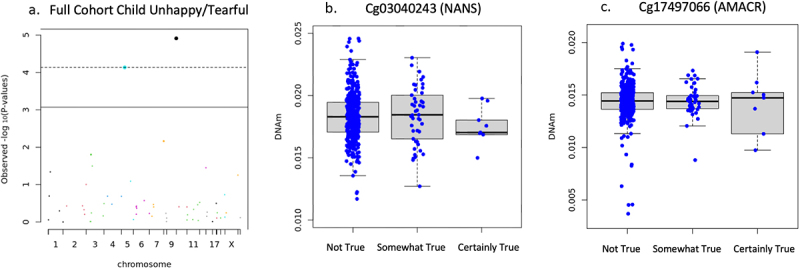
Full cohort child was unhappy/tearful in past 6 months associations.

DNAm on two CpG sites negatively associated with child had many fears in the past 6 months including cg03040243 on the body of NANS (*n* = 471, B = −.116, *t* = −4.109, FDR adjusted *p* = .001, non-adjusted *p* = 4.798 × 10^−5^), cg17497066 on the 1^st^ exon of AMACR (B = −.128, *t* = −4.102, FDR adjusted *p* = .001, non-adjusted *p* = 4.923 × 10^−5^) shown in Figure 2. DNAm of cg03040243 on the body of NANS (*n* = 473, B = −.142, *t* = −4.426, FDR adjusted *p* = .0007, non-adjusted *p* = 1.228 × 10^−5^) and cg17497066 on the 1^st^ exon of AMACR (B = −.142, *t* = −4.006, FDR adjusted *p* = .002, non-adjusted *p* = 7.319 × 10^−5^) negatively associated with child was unhappy/tearful past 6 months (see Figure 3). It is important to note that the mean DNAm reduces with ‘somewhat true,’ and slightly increases with ‘certainly true’ but the interquartile range consistently reduces as school reports of child was unhappy/tearful past 6 months increases in agreement. Newborn DNAm did not associate with child ‘adiposity rebound’ (*n* = 113), ‘child had many worries in the past six months’ (*n* = 473), or general anxiety symptom score (*n* = 809, FDR adjusted *p* > .05) on the targeted CpG sites in the full cohort.

(a) Manhattan plot of full cohort child had many fears in past 6 months and newborn DNAm. Dots on and above dotted line are FDR adjusted *p* < .05. (b) Boxplot of newborn DNAm NANS CpG site association with child had many fears in past 6 months (FDR adjusted *p* = .001). (c) Boxplot of newborn DNAm AMACR CpG site association with child had many fears in past 6 months (FDR adjusted *p* = .002).

(a) Manhattan plot of full cohort child was unhappy/tearful in past 6 months and newborn DNAm. Dots on and above dotted line are FDR adjusted *p* < .05. (b) Boxplot of newborn DNAm NANS CpG site association with child was unhappy/tearful in past 6 months (FDR adjusted *p* = .0007). The ‘certainly true’ boxplot bottom error bar is in line with the bottom of the interquartile range box. (c) Boxplot of newborn DNAm AMACR CpG site association with child was unhappy/tearful in past 6 months (FDR adjusted *p* = .002).

### Sex stratified

#### Girls

**Figure 4.**
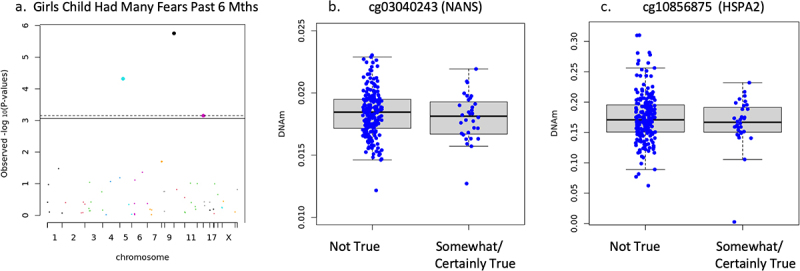
Girls only child had many fears in past 6 months associations.

**Figure 5.**
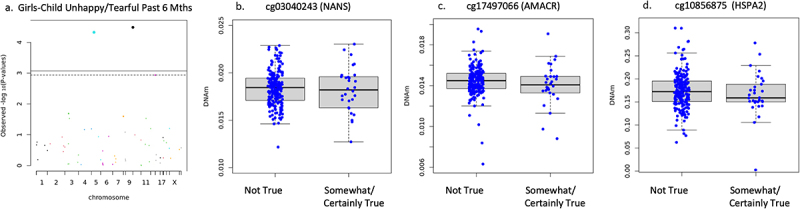
Girls only child was unhappy/tearful in past 6 months associations.

Sex-specific associations did emerge in the stratified sample. In girls, sensitivity analysis of separation anxiety showed any separation anxiety symptoms were associated but number of separation anxiety symptoms (*n* = 398) and separation anxiety symptom score (*n* = 388) did not associate. Specifically, reduced DNAm on one CpG site of the ABAT gene (cg01881182) associated with increased report of any separation anxiety symptoms (*n* = 398, B = −.199, *t* = −3.984, FDR adjusted *p* = .004, non-adjusted *p* = 8.189 × 10^−5^) but were not plotted as this variable was used for sensitivity analysis only. DNAm negatively associated with child had many fears in past 6 months in girls on cg03040243 on the body of NANS (*n* = 233, B = −.278, *t* = −4.921, FDR adjusted *p* = .0001, non-adjusted *p* = 1.756 × 10^−6^), and cg10856875 on 1^st^ exon of HSPA2 (B = −.225, *t* = −3.438, FDR adjusted *p* = .014, non-adjusted *p* = .0007) shown in Figure 4. It is important to note that cg17497066 (AMACR) also reached statistical significance, but visual observation of the boxplot showed no discernable difference in the mean or interquartile range, only the minimum and maximum observations and outliers. Therefore, the results were deemed invalid for interpretation and not reported as significant. DNAm negatively associated with child has been unhappy tearful for past 6 months on cg03040243 (NANS) (*n* = 233, B = −.230, *t* = −4.248, FDR adjusted *p* = .001, non-adjusted *p* = 3.258 × 10^−5^), cg17497066 (AMACR) (B = −.249, *t* = −4.157, FDR adjusted *p* = .001, non-adjusted *p* = 4.724 × 10^−5^), and cg10856875 (HSPA2) (B = −.204, *t* = −3.296, FDR adjusted *p* = .023, non-adjusted *p* = .001) shown in Figure 5. Child had many worries past 6 months (*n* = 233), and adiposity rebound (*n* = 54) did not associate with newborn DNAm (FDR adjusted *p* > .05) when controlling for gestational age, socioeconomic status, cell type, and ancestry in girls.

(a) Manhattan plot of targeted CpGs DNAm and child had many fears in the past 6 months in girls. Dots on and above the dotted line are statistically significant at FDR adjusted *p* < .05. (b) DNAm of NANS CpG site and child had many fears in the past 6 months in girls (FDR adjusted *p* = .0001). Variable was dichotomized due to small sample size in ‘certainly true’ category in the sex stratified sample. (c) DNAm of HSPA2 CpG site and child had many fears in the past 6 months in girls (FDR adjusted *p* = .0007).

(a) Manhattan plot of child unhappy/tearful for past 6 months for targeted CpG sites in girls. Dots on and above dotted line are significant at FDR adjusted *p* < .05. (b) NANS CpG site DNAm and child unhappy/tearful for past 6 months in girls (FDR adjusted *p* = .001). (c) AMACR CpG site DNAm and child unhappy/tearful for past 6 months in girls (FDR adjusted *p* = .001). (d) HSPA2 CpG site DNAm and child unhappy/tearful for past 6 months in girls (FDR adjusted *p* = .023).

#### Boys

**Figure 6.**
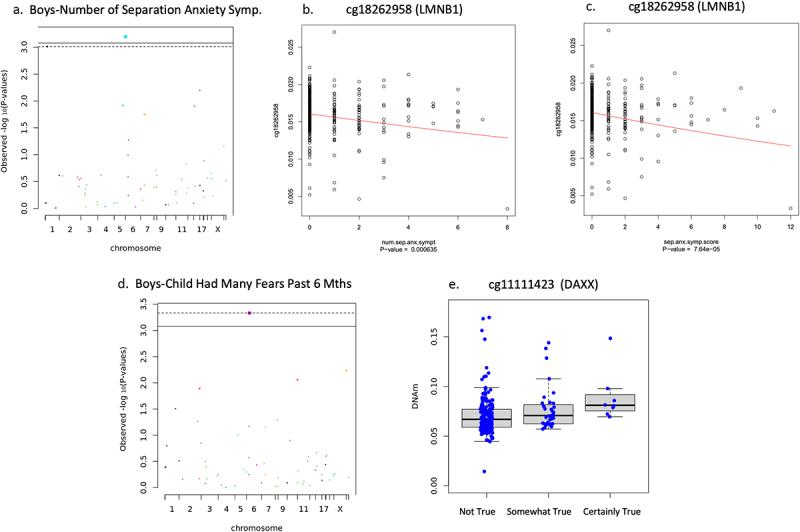
Boys’ separation anxiety and child had many fears associations.

In boys, sensitivity analysis showed newborn DNAm associations with separation anxiety varied according to how the phenotype was measured. Reduced newborn DNAm of cg18262958 on LMNB1 associated with an increased number of different separation anxiety symptoms (*n* = 417, B = −.028, *t* = −3.447, FDR adjusted *p* = .029, non-adjusted *p* = .0006), and higher separation anxiety symptom score (*n* = 403, B = −.027, *t* = −4.004, FDR adjusted *p* = .004, non-adjusted *p* = 7.640 × 10^−5^) shown in Figure 6, whereas no associations emerged with ‘any separation anxiety’ (*n* = 417, FDR adjusted *p* > .05). Newborn DNAm on cg11111423 located at 5’UTR of DAXX positively associated with ‘child had many fears in past 6 months’ (*n* = 238, B = .130, *t* = 3.560, FDR adjusted *p* = .027, non-adjusted *p* = .0004) (see Figure 6). Newborn DNAm was not associated with ‘child had many worries in past 6 months’ (*n* = 240), ‘child was unhappy/tearful for past 6 months’ (*n* = 240), ‘general anxiety symptom score’ (*n* = 415), or ‘adiposity rebound’ (*n* = 59) in boys (FDR adjusted *p* > .05).

(a) Manhattan plot of DNAm and number of separation anxiety symptoms in boys. (b) Scatterplot of LMNB1 CpG site DNAm and number of separation anxiety symptoms in boys. (c) Scatterplot of LMNB1 CpG site DNAm and separation anxiety symptom score in boys. (d) Manhattan plot of DNAm and child had many fears in past 6 months in boys. (e) Boxplot of DAXX CpG site DNAm and child had many fears in past 6 months in boys (FDR adjusted *p* = .027).

### Sex interactions

Given the within sex group unique associations among child’s mental health and cord blood DNA methylation, we tested DNA methylation and sex interaction terms with child had many fears in past 6 months, child has been unhappy/tearful for past 6 months, number of different separation anxiety symptoms, and separation anxiety symptom score. All sex interaction results are reported in Table 1. Results indicated sex moderated associations between newborn DNA methylation, on the same reported CpG sites from the sex stratified analysis (cg03040243, cg10856875, cg11111423), and child had many fears in past 6 months. One additional CpG site (cg17497066) was also associated with child had many fears that did not associate in the stratified analysis. Sex also moderated associations between DNA methylation and child was unhappy/tearful for past 6 months on the same reported CpG sites from the sex stratified analysis (cg03040243, cg17497066, cg10856875). Sex did not moderate associations between number of different separation anxiety symptoms or separation anxiety symptom score.

**Table 1.** Sex interaction results.

CpG Site	T.statistic	P.value	FDR	effect.size (B)	SE	CHR	Gene Name	Location
**Child Had Many Fears Past 6 Months**								
cg17497066	−3.7664102	0.00018991	0.00569725	−0.233605921	0.06202349	5	AMACR	1stExon
cg10856875	−3.3455682	0.000897	0.01793998	−0.251714502	0.07523819	14	HSPA2	1stExon
cg11111423	−3.1325185	0.0018578	0.02786698	−0.285589604	0.09116933	6	DAXX	5‘UTR;Body
**Child Was Unhappy/Tearful Past 6 Months**								
cg17497066	−3.8727836	0.0001252	0.00751188	−0.278594897	0.0719366	5	AMACR	1stExon
cg03040243	−3.5229705	0.00047475	0.01285143	−0.229411682	0.06511882	9	NANS	Body
cg10856875	−3.4394848	0.00064257	0.01285143	−0.300183199	0.08727563	14	HSPA2	1stExon

Sex interactions were statistically significant for child had many fears past 6 months and child was unhappy/tearful past 6 months. The same CpG sites significant for the sex interaction emerged in the stratified analysis. Number of different separation anxiety symptoms and separation anxiety symptom score did not emerge as statistically significant for a sex interaction effect.

### Child mental health mediation and moderation

**Figure 7.**
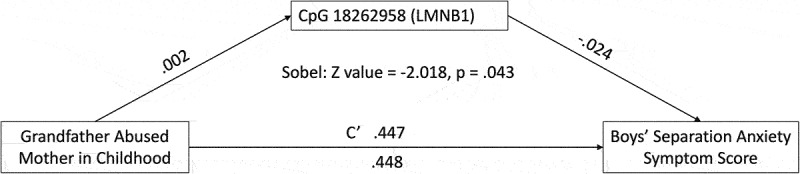
Boys’ separation anxiety symptom score mediation Model (LMNB1).

Significant associations with child outcome variables were tested for mediation (continuous dependent variables only) and moderation mechanistic action. All significant child outcome associations were tested for moderation effects. Only one mediation relationship emerged from the two continuous child outcome variables tested (number of different separation anxiety symptoms and separation anxiety symptom score). Sobel test indicated that the association between mother abused by her father in childhood and male offspring separation anxiety symptom score was mediated by DNAm of cg18262958 (LMNB1) at birth (z-score = −2.018, *p* = .043). Specifically, maternal abuse by her father in childhood was the predictor, newborn DNA methylation of LMNB1 was the mediator, and male offspring separation anxiety was the outcome. The a, b, c, and c’ pathway coefficients are shown in Figure 7. No moderation associations emerged.

Maternal physical abuse by her father in childhood was the predictor, offspring newborn DNA methylation was the mediator, and male offspring separation anxiety symptom score was the outcome variable. Sobel test indicates complete mediation by DNA methylation of LMNB1 at birth.

## Discussion

This study is the first, to our knowledge, to expand the knowledge of the mediating mechanistic roles of DNA methylation in intergenerational effects of trauma on childhood mental health. This study built upon our previous findings of epigenome-wide associations between newborn DNA methylation and maternal childhood physical abuse according to her relationship with her abuser [9]. The original findings found different biological pathways affected according to if the mother’s abuser was her mother or her father. This study examined the next step in the intergenerational pathway by investigating the connections with mothers who were abused by their fathers. Given the different biological pathways according to maternal childhood abuser relationship, delineating the mechanistic role of DNA methylation in childhood developmental outcomes required a separate examination of the intergenerational effects of mother abused by father and mother abused by mother.

The findings indicate that increased DNA methylation on the ABAT (cg01881182) and GRLX genes (cg19789021) measured in newborn cord blood, previously associated with maternal physical abuse in childhood by her father, associates with increased separation anxiety symptom scores in her child at age seven, regardless of child sex. Whereas LMNB1 (cg18262958) negatively associates with separation anxiety symptom scores. ABAT (cg01881182) also positively associated with number of different separation anxiety symptoms in the child. Our findings show that the separation anxiety symptom score is more sensitive to DNA methylation associations in newborn cord blood. It is linked to the ABAT gene, which is also associated with the number of separation anxiety symptoms in similar magnitude and direction of effects. Additionally, the separation anxiety symptom score is connected to the GRLX gene, which is not related to the number of separation anxiety symptoms. ABAT, GRLX, and LMNB1 have potential broad impacts on child development beyond the findings of this study.

The ABAT CpG site is situated in the 5’ untranslated region (5’UTR) which has been shown in previous research to exhibit an inverse relationship between DNA methylation and gene expression (Byun et al., 2012; Yin & Blanchard, 2000). This observation implies that the expression of ABAT may have been increased in these female infants at birth. ABAT plays a crucial role in the breakdown of GABA (gamma-aminobutyric acid), a significant neurotransmitter primarily involved in inhibitory functions within the central nervous system (GeneCards, 2023). ABAT facilitates the conversion of GABA into succinic semialdehyde. It is estimated that GABA is found in around 33% of synapses in the central nervous system (Llewellyn-Smith et al., 1995). Multiple clinical presentations are associated with ABAT deficiency affecting psychomotor activity (psychomotor retardation), muscle functioning (hypotonia, hyperreflexia), and abnormalities shown in electroencephalogram (EEG) readings (GeneCards, 2023).

GLRX is another important consideration. A member of the glutaredoxin family is encoded by GLRX. The encoded protein functions as a cytoplasmic enzyme that facilitates the reversible reduction of glutathione-protein mixed disulphides. This enzyme plays a significant role in the antioxidant defence system. Controlling the S-glutathionylation status of signalling mediators plays a vital role in multiple signalling pathways. The pathways associated with it include the PAK pathway and the metabolism of nucleotides. Typically, the transcription start site is considered to be the necessary location for methyl groups to silence transcription of the gene resulting in downregulation. However, DNA methylation of the gene body has shown a positive correlation with gene expression (Aran et al., 2011; Ball et al., 2009; Hellman & Chess, 2007; Laurent et al., 2010; Lister et al., 2009). Yet, research is still evolving and indicates that DNA methylation at the first exon is more likely to regulate transcription of the gene (Brenet et al., 2011), and the link between gene-body DNA methylation and expression levels is bell-shaped (Jjingo et al., 2012). Intermediate level expressed genes have the most gene-body methylation, while low- and high-expressed genes have low levels (Jjingo et al., 2012). The augmented DNA methylation seen at the GLRX body CpG site may potentially serve as a marker for moderate levels of gene expression. However, the specific biological consequences resulting from this phenomenon remain uncertain.

The gene LMNB1, also known as Lamin B1, is classified as a protein-coding gene. Two of the linked pathways associated with LMNB1 are the apoptosis and survival FAS signalling cascades, as well as the Defective Intrinsic Pathway for Apoptosis (GeneCards, 2023). Reduced DNAm at the transcription start site suggests potential increased LMNB1 genetic expression. Of interest, LMNB1 has been linked to neuronal migration in knockout mice models whereupon absence of lamin B1 or lamin B2 resulted in serious neurodevelopmental abnormalities [32]. Impaired neuronal migration has been linked to cognitive deficits in mice and is theorized to explain cognitive deficits in extremely preterm infants [33]. The suspected upregulation (reduced DNAm at TSS) of LMNB1 in relation to increased separation anxiety symptom scores presents a challenge to extrapolating meaning. However, the complexity of gene interactions may be at play between ABAT and LMNB1 to explain the findings. Gaba, the neurotransmitter that ABAT plays a role in regulating, has been shown to moderate neuronal migratation as well. Inhibited gaba release from endothelial cells, in mice, was shown to impair migration of cortical interneurons resulting in a novel theory of the aetiology of neuropsychiatric disorders such as anxiety [34]. Moreover, gaba and glutamate exist in balance and this balance is disrupted in anxiety disorders [35]. It is possible that the suspected downregulation of ABAT (increased DNAm at 5’UTR) disrupts a healthy balance of gaba and glutamate. Thereby, the epigenetic regulation of LMNB1 could be in response to the changes in gaba signalling.

NANS and AMACR both showed negative associations with child had many fears and was unhappy/tearful in the past 6 months. We were unable to identify any literature linking NANS or AMACR to anxiety or depression symptoms. However, candidate gene analysis identified AMACR in association with schizophrenia [36]. Therefore, our findings are the first to our knowledge to identify these associations. NANS encodes an enzyme involved in the biosynthesis of sialic acids. In laboratory conditions, the protein produced by NANS uses N-acetylmannosamine 6-phosphate and mannose 6-phosphate as substances it acts upon to produce phosphorylated forms of N-acetylneuraminic acid (Neu5Ac) and 2-keto-3-deoxy-D-glycero-D-galacto-nononic acid (KDN). However, it shows significantly greater activity towards the Neu5Ac phosphate product [37]. The related pathways are Synthesis of substrates in N-glycan biosynthesis and Metabolism of proteins. The AMACR enzyme catalyzes the conversion of pristanoyl-CoA and C27-bile acylCoAs between their (R)- and (S)-stereoisomers [37]. The transformation to the (S)-stereoisomers is essential for the breakdown of these substrates through peroxisomal beta-oxidation. The proteins derived from this genetic location are distributed in both peroxisomes and mitochondria. The related pathways of interest include the synthesis of bile acids and bile salts, as well as peroxisomal lipid metabolism. It is unclear, at this time, what biological consequences may occur in relation to the NANS and AMACR epigenetic differences.

The sex-stratified results showed two distinct relationships. First, separation anxiety only associated with DNAm in boys. Second, sex-stratified newborn DNAm revealed different genes with different effects associated with child had many fears in past 6 months. Specifically, HSPA2 (cg10856875) was negatively associated in girls and DAXX (cg11111423) was positively associated in boys. HSPA2 plays a role in inhibiting the formation of inclusion bodies and the process of protein refolding. Cellular responses to stimuli and meiosis are two of its related pathways (GeneCards, 2023). HSPA2 has previously been theorized to play a role in regulating corticosterone-induced stress effects on immune function in chickens [38], but no human or closely related animal model studies could be identified. A multifunctional protein that is encoded by DAXX is found in various parts of the cytoplasm and nucleus. Within the cytoplasm, the protein that has been encoded may serve the purpose of regulating apoptosis. Two related pathways are gene expression and deficient inhibition of DNA recombination at the telomere caused by ATRX mutations [37]. Benes [39] identified DAXX as one of the genes that could potentially have a significant impact on regulating the activity levels of GABA neurons during early adulthood. This influence is mediated by each of the genes’ level of functional specialization and the integrity of the SO-CA3/2 interneurons at the genomic level.

The sex interaction tests revealed that DNA methylation associations with separation anxiety variables were not moderated by sex, while the child fear and unhappiness variables were statistically significant. Separation anxiety symptom score (girls: *M* = .74, SD = 1.84; boys: *M* = .72, SD = 1.68) and number of different separation anxiety symptoms (girls: *M* = .65, SD = 1.42; boys: *M* = .65, SD = 1.34) did not statistically differ by sex (T-test *p* > .05). The sample size for these variables was also similar among child sex (girls = 398, boys = 417). While there were no differences between sex groups for cord blood DNA methylation associations with separation anxiety variables, boys showed a within group statistically significant association. Our findings suggest that sex moderation (between groups) and sex-stratified (within group) analyses may reveal different findings relevant for understanding sex effects. This may be critical for cord blood DNA methylation investigations given the different endocrine environments in which male and female foetuses develop.

The observed variations in the effects of sex indicate potential moderating influences of the endocrine system or sex-specific disparities in DNA methylation functionality during prenatal development. Divergent endocrine circumstances during foetal development in relation to sex has been observed [40]. Additionally, it has been shown that male foetuses have heightened DNA methylation activity during the masculinization of the male brain [41]. The DNA methylation process implicated in the masculinization of the male brain may potentially enhance male susceptibility to prenatal influences, such as changes in maternal allostatic load or stress responsivity resulting from mother’s childhood trauma. This observation may provide an explanation for the separation anxiety associations unique to boys in this sample. Furthermore, we have shown that neonatal DNA methylation plays a mediation role in the link between maternal childhood physical abuse by her father and the mental health functioning of her child at the age of seven. Moreover, this mediating association occurred in the specific context of maternal childhood abuse, namely physical abuse perpetrated by the father as opposed to the mother or any other individual. Additionally, this association was shown to be sex-specific affecting male offspring only. The potential influence of DNA methylation on developmental outcomes in children has been a subject of interest for a considerable period of time [42–44]. Our research findings provide further support for the importance of continued investigation into the role of DNAm in the transmission of trauma effects across generations. Moreover, the inclusion of maternal trauma effects, the mechanistic involvement of DNAm in children, and the sex-specific relationships provide supplementary elucidation for the disparities observed in trauma-related outcomes, particularly in the intergenerational transmission of trauma.

It is important to acknowledge the limitations of this study when interpreting the results. The study cohort’s demographics impose limitations on the generalizability of the findings. The predominant demographic within the cohort consisted of individuals who self-identified as European-American (Caucasian) and were reported to belong to the middle socioeconomic class. To mitigate the potential influence of ancestry (race) and socioeconomic status (SES) on our study outcomes, we controlled for race and SES in all models. Nonetheless, it is worth noting that there may exist connections unique to ancestry and socioeconomic factors that were beyond the scope of our analysis. Furthermore, authors did not have data on mother’s smoking status and therefore were unable to control for this factor. However, previous findings on this cohort did not show maternal smoking and DNA methylation associations with the CpG sites or genes indicated in the current study [45]. In order to enhance the scope of future study, it is recommended that purposeful sampling be employed to obtain cohorts that exhibit a wider range of ancestral backgrounds and socioeconomic status, and that maternal smoking status is considered.

## Conclusion

The objective of this study was to examine the subsequent impact on child developmental outcomes, encompassing mental health and physical growth, from previously identified DNAm in newborns that associated with maternal history of physical abuse by fathers. Additionally, the study aimed to investigate whether newborn DNAm played a mediating or moderating role in these developmental outcomes. The DNAm of newborns shown to be associated with the maternal experience of physical abuse inflicted by her father during her own childhood, is in turn linked to the mental well-being of the child. Sex-specific effects have been observed for direct effects, and infant DNAm plays a role in mediating the influence of intergenerational trauma on the development of mental health in children, with these effects being specific to males. No significant DNAm moderating associations were detected.

## Data Availability

The data that support the findings of this study are available from the Avon Longitudinal Study of Parents and Children (ALSPAC). Restrictions apply to the availability of these data, which were used under licence for this study.
